# Association Between the Use of Oral Contraceptives and the Development of Rheumatoid Arthritis: A Systematic Review and Meta-Analysis

**DOI:** 10.3390/jcm14082710

**Published:** 2025-04-15

**Authors:** Annalisa Marino, Damiano Currado, Onorina Berardicurti, Marta Vomero, Lyubomyra Kun, Letizia Pia Di Corcia, Erika Corberi, Francesca Trunfio, Francesca Saracino, Ludovica Lamberti, Leonardo Frascà, Angelo Battista, Marta Alfano, Manuela Pietramale, Silvia Schiavone, Roberto Giacomelli, Luca Navarini

**Affiliations:** 1 Clinical and Research Section of Rheumatology and Clinical Immunology, Fondazione Policlinico Universitario Campus Bio-Medico, 00128 Rome, Italy; a.marino@policlinicocampus.it (A.M.); o.berardicurti@policlinicocampus.it (O.B.); r.giacomelli@policlinicocampus.it (R.G.); l.navarini@policlinicocampus.it (L.N.); 2 Rheumatology and Clinical Immunology, Department of Medicine, University of Rome “Campus Biomedico”, School of Medicine, 00128 Rome, Italy; m.vomero@unicampus.it (M.V.); lyubomyra.kun@unicampus.it (L.K.); letiziapia.dicorcia@unicampus.it (L.P.D.C.); erika.corberi@unicampus.it (E.C.); francesca.trunfio@unicampus.it (F.T.); francesca.saracino@unicampus.it (F.S.); ludovica.lamberti@unicampus.it (L.L.); leonardo.frasca@unicampus.it (L.F.); battista880@gmail.com (A.B.); marta99.allfano@gmail.com (M.A.); manuelapietramale@gmail.com (M.P.); silviaschiavone00@libero.it (S.S.)

**Keywords:** rheumatoid arthritis, oral contraceptives, meta-analysis, hormonal factors, risk assessment

## Abstract

**Background:** Rheumatoid arthritis (RA) is a chronic inflammatory joint disease that significantly impacts quality of life, particularly among women. Previous studies have suggested that oral contraceptive (OC) use may influence RA risk, but conflicting findings from earlier meta-analyses necessitate an updated analysis incorporating more recent data. **Methods:** We conducted a systematic review and meta-analysis of observational studies on OC use and RA risk by searching MedLine (via PubMed), Scopus, and Cochrane Databases up to September 2024. **Results:** Our analysis demonstrated that current or prior use of OCs is associated with a statistically significant reduction in RA risk (OR 0.80, 95% CI 0.70–0.91). In contrast, the associations for current use (OR 0.59, 95% CI 0.34–1.02) and past use (OR 0.83, 95% CI 0.69–1.01) were less definitive, likely due to substantial heterogeneity among studies. Cumulative meta-analysis revealed a modest temporal trend toward a protective effect of OC use. **Conclusions:** This meta-analysis supports a protective association between current or prior OC use and the development of RA, highlighting the potential role of hormonal factors in RA pathogenesis.

## 1. Introduction

Rheumatoid arthritis (RA) is a chronic inflammatory joint disease characterized by a distinct pattern of joint degradation, posing a significant threat of loss of function and mobility if left untreated [1]. This disease results in partial or permanent disability in the majority of affected individuals and is characterized by persistent synovitis and pannus formation—a proliferative synovial tissue that eventually drives the degradation of cartilage, subchondral bone, and periarticular soft tissues [2,3].

Its prevalence in Europe and the United States is estimated to range between 0.5% and 1.0%, with women exhibiting greater susceptibility than men [4,5]. The correlation between RA and hormonal levels, specifically estrogen and progesterone, has been a subject of ongoing investigation, in particular regarding serological markers and the signs and symptoms associated with the development of the disease [6].

Oral contraceptives (OCs), commonly used for birth control or in hormone replacement therapies, predominantly consist of estrogen and progesterone [7].

Hormonal and reproductive factors contribute to our understanding of rheumatoid arthritis risk [8].

Breastfeeding, according to the literature, has been associated with a reduced risk of RA, while an early age at menopause has been reported to confer a higher risk of RA, particularly the seronegative subset. Simultaneously, lower testosterone levels in men are associated with an increased risk of seronegative RA [9,10].

The relationship between OCs and RA is probably driven by estrogen. In fact, estrogen appears to suppress cell-mediated immune responses, reduce the secretion of proinflammatory cytokines, and prevent the activation of osteoclasts. Collectively, these effects decrease synovial pannus formation and reduce the severity of signs and symptoms associated with RA [11].

Previous systematic reviews suggested an inverse relationship between the use of oral contraceptives and the onset of RA, potentially explaining the recent decline in the incidence of RA among women over the past 50 years due to increased oral contraceptive use [12,13]. Although three meta-analyses in the 1990s explored the association between oral contraceptive use and the development of RA, inconsistencies in findings resulted from the limited inclusion of studies. A more recent meta-analysis from 2014 concluded that OC use has no protective effect on RA onset but appears to prevent progression to severe RA [14]. Since 2014, however, new population studies have been conducted, with important data and many patients that can be included to perform a precise and timely analysis.

This systematic review and meta-analysis aim to define an evidence-based strategy to understand whether estrogen use is related to the risk of RA development by analyzing observational clinical studies of cohorts of RA patients who use OCs published from the 1990s to the present.

## 2. Materials and Methods

### 2.1. Protocol

**Figure 1:**
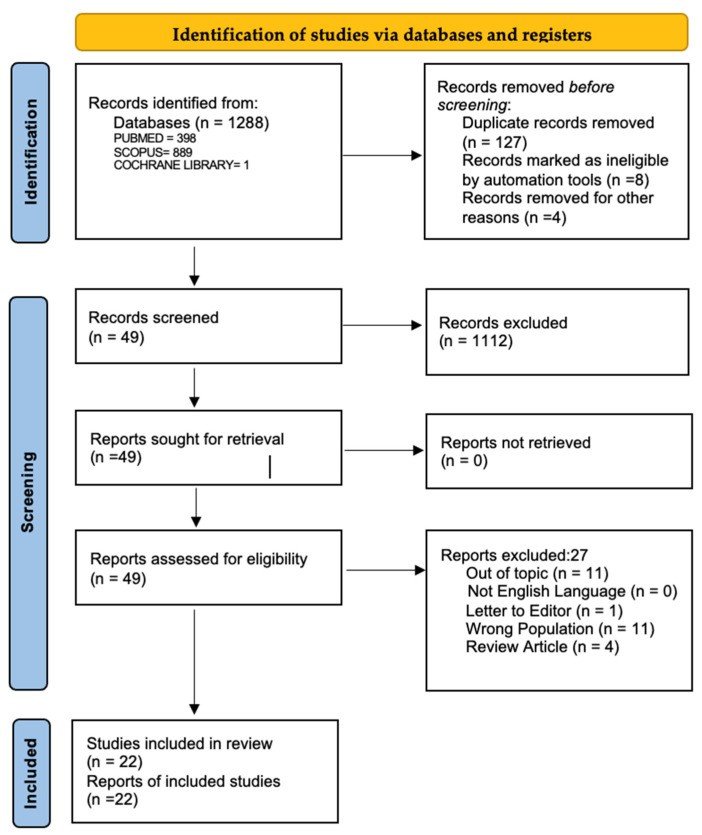
PRISMA 2020 flow diagram showing an overview of the study selection process. Source: [15].

This study was conducted according to the Cochrane Collaboration and the Preferred Reporting Items for Systematic Reviews and Meta-Analyses (PRISMA) statement, as shown in Figure 1. It also conforms with the guidelines of Meta-Analyses and Systematic Reviews of Observational Studies (MOOSE) [15].

The PRISMA-P and MOOSE checklists are presented as Supplementary Tables S1 and S2, respectively.

### 2.2. Search Strategy

We systematically searched MedLine (via PubMed), Scopus, and Cochrane Databases up to September 2024. For the research, we used the string (“oral contraceptive” OR “OCs” OR “estrogen-progestin” OR “estroprogestinic” OR “HRT” OR “oral contraception” OR “hormone replacement therapy” OR “birth control pill” OR “contraceptive pill” OR “oral contraceptive agent”) AND (“rheumatoid arthritis” OR “RA”). In addition, relevant keywords were used in different combinations for freehand search, and the bibliography of the selected articles was revised to improve the search strategy’s sensitivity, as shown in Figure 1.

The PICO question (population, intervention, comparison, outcome) was the following:

- Population: all the RA adult population;
- Intervention: current or prior use of OCs;
- Comparator: no current or prior use of OCs;
- Outcomes: odds ratio (OR) and 95% confidence interval (CI) for the association between OC use and the risk of RA development.

Publications written in a language other than English were excluded.

### 2.3. Eligibility Criteria

For the primary search, based on preliminary scouting, we included all clinical studies reporting results regarding the use of OCs in RA patients.

### 2.4. Data Extraction and Quality Assessment

Data were extracted and summarized by 4 independent reviewers (A.M, A. B, M.A, M.P., S.S.) and verified by 2 senior reviewers (L.N, O.B). From each selected article, the following information was collected: first author, year of publication, origin, study design, total number of patients, and use of OCs.

The authors were contacted to obtain necessary information when data were missing, incomplete, or inconsistent. No protocol for this systematic review was registered in any international registry.

The quality of the studies included in the quantitative analysis was assessed using the “star system” of the Newcastle–Ottawa Quality Assessment Scale (NOS) [16]. The score ranges from 0 to 9 stars. Studies that scored ≥ 7 stars were considered high-quality. For case series studies, we evaluated the quality using the Quality Assessment Tool for Before–After (Pre–Post) Studies with No Control Group proposed by the National Heart, Lung, and Blood Institute—US Department of Health and Human Services (https://www.nhlbi.nih.gov/health-pro/guidelines/in-develop/cardiovascular-risk-reduction/tools/before-after, accessed on 1 December 2022). Quality evaluation was performed independently by two reviewers (D.C. and M.V). If there was any disagreement in the scores, a third reviewer (L.P.D.C) was involved to re-evaluate the original study.

Additionally, funnel plots were employed to visually assess publication bias (Supplementary S3).

### 2.5. Statistical Analysis

The relationship between the use of OCs and the risk of RA development was assessed using OR and 95% confidence intervals. Significant heterogenicity was expected among studies, so data were combined using a random effect model. The Cochrane chi-square (Cochrane Q) test and I2 test were carried out to analyze the heterogenicity among the results of different studies. An I2 test < 25% indicated no heterogenicity, while I2 > 50% and/or *p* < 0.05 indicated substantial heterogenicity [17].

The Leave-One-Out method was used to evaluate if a single study exerted a very high influence on the overall results. The Egger test and a funnel plot were used to assess the publication bias [18]. Moreover, a cumulative metanalysis was reported to detect the modification of estimates according to the publication date. All the analyses were performed using RStudio (v. 1.2.5042, the R Foundation for Statistical Computing, Vienna, Austria) and the Metafor package (https://www.metafor-project.org/doku.php/metafor, accedes on 10 January 2025), and all the statistical steps were supervised by 3 statistician reviewers (L.N, D.C, O.B.).

## 3. Results

### 3.1. Study Selection and Characteristics

Using the search strategy, 2014 peer-reviewed articles were retrieved. After the first screening of titles and abstracts, 280 articles were selected for full-text assessment. After review, 22 studies were included in the qualitative and quantitative analysis, divided into subgroups considering current and past use of OCs, and whether the participants had ever used OCs. The protective effect of OC use on RA onset was seen in case–control studies, nested case–control studies, cohort studies, and prospective cohort studies.

First of all, we analyzed whether there had been any use of estrogens, i.e., RA patients who had taken oral estrogen replacement therapy at some point in their life.

Among these studies, three were conducted in the Netherlands [19,20,21], five in Sweden [22,23,24,25,26], one in Lebanon [27], one in Greece [28], three in the United Kingdom [29,30,31], two in France [32,33], one in Canada [34], three in the USA [35,36,37], one in Denmark [38], one in China [39], and one in South Korea [40].

The main characteristics of the selected studies for the use of OCs are reported in Table 1. For current use, defined as individuals who were actively taking oral contraceptives at the time of data collection, the following studies were analyzed, as reported in Table 2.

Among these, four were conducted in Sweden, i.e., [22,25,26,41], three in the USA [36,42,43], and two in the United Kingdom [29,30].

For past use, defined as individuals who had previously used oral contraceptives but discontinued them before the time of data collection, the following studies were analyzed, as reported in Table 3. Among these, two were conducted in Sweden [22,26], two in the USA, [43,44], and two in the United Kingdom [45,46].

**Table 1:** Overview of the selected studies on OC use at any point and RA risk. OR = odds ratio; LL = lower limit; UL = upper limit.

USE OF OCs Ever									
ID	First Author	Year	Country	Age	Cases	Controls	OR	LL	UL
1	Vandenbroucke [19]	1982	Netherland	25–54	228	302	0.54	0.34	0.86
2	Allebeck P [22]	1984	Sweden	15–50	76	152	0.58	0.41	0.80
3	Vandenbroucke [20]	1986	Netherland	18–90	246	323	0.57	0.32	1.00
4	Darwish MJ [27]	1987	Lebanon	18–90	86	86	1.29	0.64	2.58
5	Linos A [28]	1989	Greece	>20	135	104	0.76	0.22	2.70
6	Spector T.D. [31]	1990	UK	35–70	150	180	0.60	0.30	1.17
7	Van Zeben D. [21]	1990	Netherland	20–50	222	378	0.44	0.27	0.71
8	Hazes JM [29]	1990	UK	18–90	94	323	0.39	0.24	0.62
9	Silman A [30]	1992	UK	18–40	88	144	0.82	0.42	1.60
10	Jorgensen C [32]	1996	France	25–84	176	145	0.54	0.34	0.85
11	Pope JE [34]	1999	Canada	19–44	34	68	0.60	0.10	3.10
12	Merlino LA [35]	2003	USA	55–69	32	126	1.00	0.67	1.50
13	Doran MF [36]	2004	USA	≥18	50	70	0.57	0.35	0.91
14	Karlson EW [37]	2004	USA	30–55	304	354	0.74	0.59	0.92
15	Pedersen M [38]	2006	Denkmark	18–65	365	477	1.24	0.91	1.71
16	Pikwer M. [23]	2009	Sweden	44–74	130	514	1.03	0.63	1.67
17	Berglin [24]	2010	Sweden	20–69	70	280	0.79	0.45	1.38
18	Adab P [39]	2014	China	20–69	669	7439	1.18	0.84	1.67
19	Orellana C [25]	2015	Sweden	50	467	935	0.90	0.70	1.20
20	Orellana C [26]	2017	Sweden	60.8–61	2578	4129	0.87	0.78	0.97
21	Eun Y [40]	2020	South Korea	≥40	5759	1,284,676	0.99	0.93	1.07
22	Salliot C [33]	2021	France	18–90	681	75,397	1.17	1.01	1.37

**Table 2:** Overview of the selected studies on OC current use and RA risk. OR = odds ratio; LL = lower limit; UL = upper limit.

CURRENT USE OF OCs									
ID	First Author	Year	Country	Age	Cases	Controls	OR	LL	UL
1	Allebeck P [22]	1984	Sweden	15–50	15	22	0.71	0.27	1.87
2	Koepsell T [42]	1989	USA	18–64	141	36	0.27	0.06	0.97
3	Hazes JMV [29]	1990	UK	20–50	39	91	0.58	0.32	1.04
4	Hernandez-Avila M [43]	1990	USA	30–55	2	72	1.30	0.30	6.50
5	Silman A [30]	1992	UK	18–40	15	41	0.51	0.22	1.19
6	Reckner Olsson A [41]	2001	Sweden	25–75	179	259	0.30	0.70	0.80
7	Doran MF [36]	2004	USA	≥18	45,209	45,209	1.00	0.40	2.52
8	Orellana C [25]	2015	Sweden	60.8–61	40	105	0.70	0.40	1.00
9	Orellana C [26]	2017	Sweden	≥18	195	331	0.85	0.68	1.06

**Table 3:** Overview of the selected studies on OC past use and RA risk. OR = odds ratio; LL = lower limit; UL = upper limit.

PAST USE OF OCs									
ID	First Author	Year	Country	Age	Cases	Controls	OR	LL	UL
1	Allebeck P [22]	1984	Sweden	15–50	7	34	0.39	0.24	0.63
2	Del Junco DJ [44]	1985	USA	24–68	182	182	1.00	0.60	1.70
3	Hannaford PC [45]	1989	UK	18–90	151	125	0.94	0.72	1.22
4	Hernandez-Avila M [43]	1990	USA	30–55	41	72	0.90	0.60	1.30
5	Camacho EM [46]	2011	UK	16–50	184	339	0.92	0.62	1.36
6	Orellana C [26]	2017	Sweden	18–90	1522	2531	0.87	0.78	0.98

### 3.2. OC Use at Any Point, Risk of RA, and Cumulative Meta-Analysis

**Figure 2:**
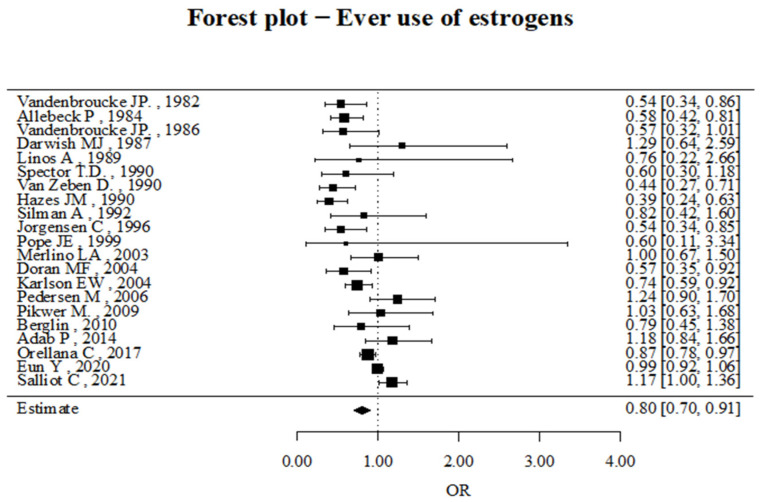
Forest plots of odds ratio (OR) of ever having used OCs and RA risk [19,20,21,22,23,24,26,27,28,29,30,31,32,33,34,35,36,37,38,39,40].

As shown in Figure 2, a protective role of having ever used OCs concerning RA development was observed (OR 0.8, 95% CI 0.7–0.91).

**Figure 3:**
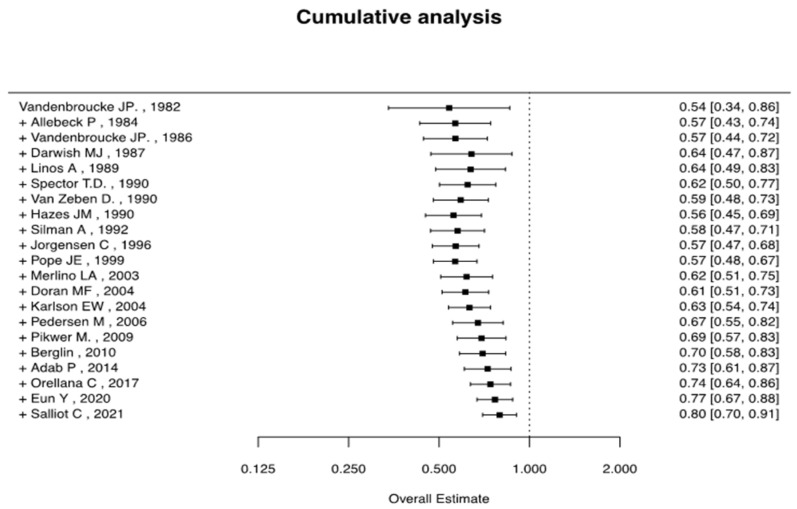
Cumulative analysis of the use of OCs at any point and RA risk [19,20,21,22,23,24,26,27,28,29,30,31,32,33,34,35,36,37,38,39,40].

In the cumulative meta-analysis examining the risk of RA associated with using OCs, a temporal trend emerges, indicating a tendency toward a protective effect, as shown in Figure 3. However, this temporal effect is modest, as evidenced by the gradual convergence of the OR values toward the midline.

### 3.3. OC Current Use, Risk of RA, and Cumulative Meta-Analysis

Few studies have examined current use of OCs, and there is significant heterogeneity (I2 (total heterogeneity/total variability): 92.49%).

**Figure 4:**
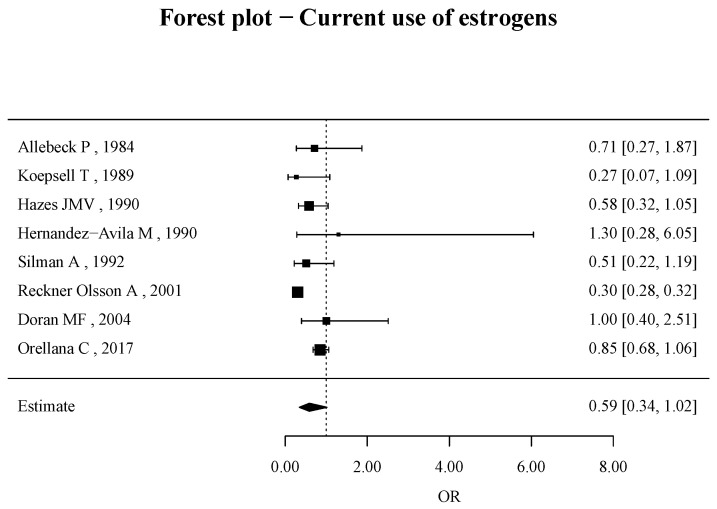
Forest plots of odds ratio (OR) for current use of OCs and RA risk [22,26,29,30,36,41,42,43].

The risk of RA development with current oral contraceptive use is 0.59 (95% CI, 0.34–1.02), as shown in Figure 4.

**Figure 5:**
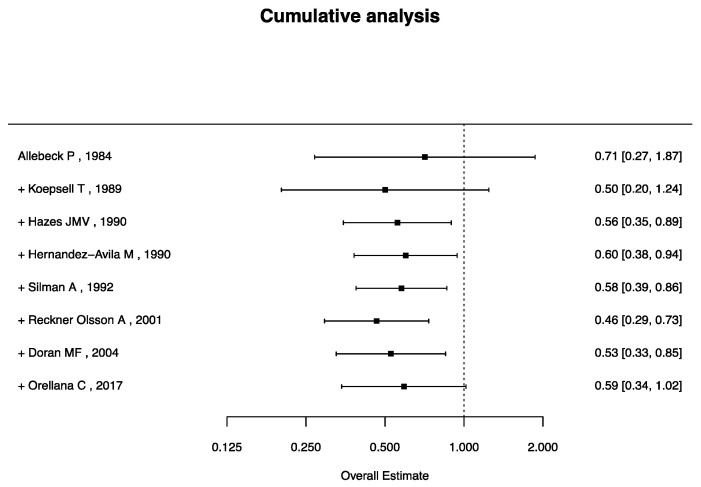
Cumulative analysis of current use of OCs and RA risk [22,26,29,30,36,41,42,43].

The results of the cumulative metanalysis on the current use of OCs are show in Figure 5: while the cumulative analysis suggests a potential protective effect of current OC use, the overall estimate remains borderline and does not reach statistical significance, highlighting residual uncertainty in the association.

### 3.4. OC Past Use, Risk of RA, and Cumulative Meta-Analysis

**Figure 6:**
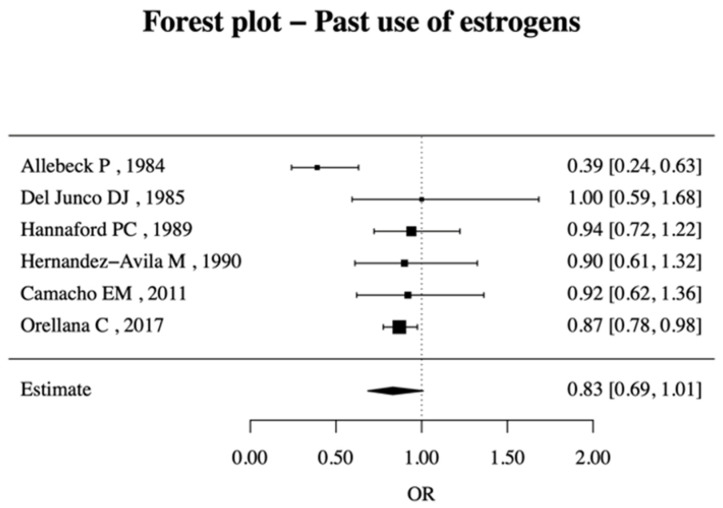
Forest plot of past use of OCs and RA risk [22,26,43,44,45,46].

**Figure 7:**
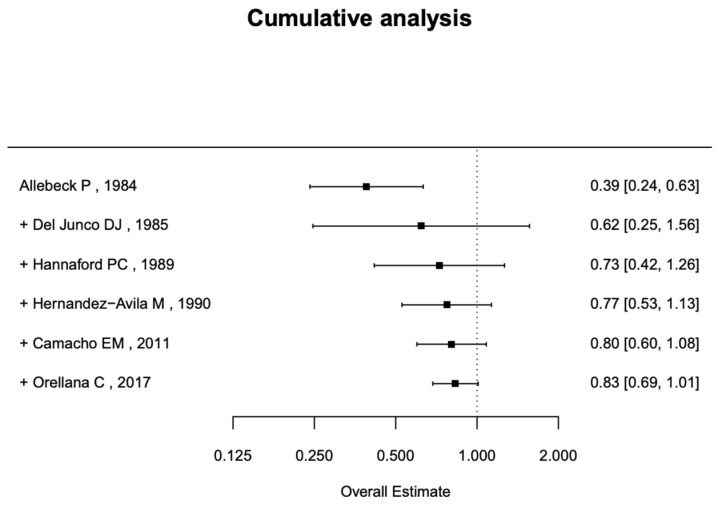
Cumulative analysis of past use of OCs and RA risk [22,26,43,44,45,46].

Few studies (only six) have examined the past use of estrogens. The risk of RA development associated with past use is 0.83 (95% CI, 0.69–1.01), as shown in Figure 6. While some individual studies suggest a potential protective effect, the overall estimate does not reach statistical significance, indicating uncertainty in the association. (Figure 7).

### 3.5. Publication Bias

The publication bias was evaluated with a different funnel plot. For use at any point, the Egger’s test was used to test funnel plot asymmetry, and the analysis showed t = −2.4668, df = 19, and *p* = 0.0233, not suggesting asymmetry (Figures in Supplementary Materials).

For current use, the Egger’s test was used to test funnel plot asymmetry, and the analysis showed t = 1.8140, df = 6, and *p* = 0.1196. For past use, the Egger’s test was used to test funnel plot asymmetry, and the analysis showed t = −0.5716, df = 4, and *p* = 0.5982. In both cases, this showed an important asymmetry.

To further assess potential publication bias, especially in the current and past use subgroups, we applied the trim-and-fill method. For current use, the original pooled OR was 0.59 (95% CI, 0.34–1.02), which shifted to 0.68 (95% CI: 0.44–1.04) after adjustment. For past use, the OR changed from 0.83 (95% CI: 0.69–1.01) to 0.89 (95% CI: 0.72–1.10) following correction. These adjustments suggest a potential, though limited, impact of publication bias—more evident in the current use subgroup. Nonetheless, the overall interpretation of the findings remains robust. Funnel plots with trim-and-fill correction are available in the Supplementary Materials.

## 4. Discussion

In this meta-analysis, ever having used OCs was confirmed to be a protective factor against the development of RA, considering recent population studies published in the last 10 years [14,32,47].

The predominance of females being affected by different autoimmune diseases, including RA, suggests the potential role of sex hormones and reproductive factors in both the development and severity of RA [44]. Women with a lower age at menarche demonstrate a comparatively reduced risk of developing RA [48,49].

The previous literature did not conclusively indicate an impact of OCs on the risk of RA development or whether OCs play a protective role [14,32]. Therefore, this article analyzes available data from observational studies published between 1982 and the present, focusing on cohorts of patients with rheumatoid arthritis who have used OCs.

This study incorporates available published observational studies, including 12,640 patients with RA. Our systematic review and meta-analyses show that an inverse association was found between the habitual use of oral contraceptives and the development of RA in case–control studies, demonstrating a protective effect of OC use at any point compared to no use of OCs in the same patient cohort [50].

The use of OCs, characterized by their composition of estrogen and progesterone, is postulated to influence the development and severity of RA through immunomodulatory and anti-osteoclastic mechanisms [51]. These potential effects highlight the intricate interplay between hormonal factors and RA pathogenesis. Firstly, one hypothesis posits that estrogen, a key component of OCs, may exert immunomodulatory effects by suppressing cell-mediated immune responses [52]. This suppression is believed to reduce the secretion of proinflammatory cytokines, ultimately leading to the amelioration of RA symptoms. Secondly, an alternative proposed mechanism suggests that estrogen in OCs may impede the formation of osteoclasts and synovial pannus. This inhibition is anticipated to mitigate the extent of bone destruction associated with RA [53].

Several studies using mouse models have also confirmed that estrogen reduces susceptibility to RA development [54,55]. Using mice genetically prone to developing RA (DBA/1 strain), Nilsson et al. found that the disease occurs more readily in animals that were ovariectomized and exposed to low concentrations of antigen and type II collagen (CII). Furthermore, the number and percentage of B220 + CR1 + (complement receptor 1) B-cells in the lymph nodes, spleen, and peripheral blood, as well as CR1 expression, were decreased in these mice; therefore, estrogens could increase CR1 expression. The protective effect is dependent on the CII dose because CR1 can only reduce inflammation when mice are treated with low doses of antigen (CII) but can arouse an immune response with higher doses of antigen [56].

Therefore, the protective effect of estrogens and progesterone is dependent on the antigen dose, as might occur in hormonal formulations. Furthermore, combined treatment with dexamethasone plus estrogen in these same mice (ovariectomized DBA/1) reduced B-cell frequency, autoantibody concentration, and inflammation incidence (IL6), reducing disease manifestations, cartilage destruction, and osteoporosis. The same results were obtained with dexamethasone plus raloxifene, a selective estrogen receptor modulator (SERM) [57].

It is known that in RA, the presence of autoantibodies precedes the inflammatory phase; the sialylation status of these antibodies is important in this disease. Low sialylation of IgG has been correlated with the progression of inflammation, while high sialylation of IgG reduces disease manifestations. High levels of estrogen have been reported to increase the expression of Beta-galactoside alpha-2,6-sialyltransferase 1 (ST6GAL1), the enzyme responsible for sialic acid binding to IgG, thereby increasing IgG sialylation and possibly inducing an anti-inflammatory effect in RA patients [58]. Therefore, estrogen may have a protective effect on RA by reducing inflammation. Furthermore, as far as T helper cells are concerned, estrogen modulates their polarization, shifting the immune response from a proinflammatory T helper type 1 (Th1) phenotype to a more regulatory T helper type 2 (Th2) phenotype. In addition, estrogen enhances the development and suppressive function of regulatory T cells (Treg), thus promoting immune tolerance [59]. Moreover, there are several mechanisms in which these hormones are implicated; with regard to osteoclastogenesis, estrogen promotes osteoclast apoptosis and inhibits osteoclastogenesis by stimulating Osteoprotegerin (OPG) production and reducing osteoclast differentiation after the suppression of Interleukin-1 (IL-1) and Tumor Necrosis Factor (TNF) and the subsequent release of Macrophage Colony-Stimulating Factor (M-CSF), Receptor Activator of Nuclear Factor κB Ligand (RANKL), and Interleukin-6 (IL-6). It can also activate the Wingless/Integrated (Wnt) Signaling Pathway to increase osteogenesis and increase Bone Morphogenetic Protein (BMP) signaling to promote the differentiation of mesenchymal stem cells from pre-osteoblasts to osteoblasts instead of adipocytes. Estrogen deficiency leads to the uncoupling of bone resorption and formation; as a result, increased bone loss occurs [60,61].

Therefore, further studies are required to better understand how different timings of OC use may affect RA development risk.

Building on this biological rationale, we conducted a cumulative analysis of the available evidence to better understand the clinical impact of hormonal influences in RA. In our study, from the earliest available study (Vandenbroucke JP, 1982 [19]) to the most recent (Salliot C, 2021 [33]), each eligible study was added sequentially to conduct a cumulative analysis. Regarding the analysis of current and past use of oral contraceptives, we had a much lower number of studies with very significant heterogeneity. This is demonstrated by our highly asymmetric funnel plots, which do not allow us to draw appropriate conclusions, indicating significant publication bias. Several factors may contribute to this variability. First, the data analyzed originate from observational studies characterized by differing baseline features and recruitment criteria. Specifically, for the current use of OCs and past use of OCs, we had a limited number of studies, and each included cohorts defined by different RA classification criteria at different time points. In particular, from 1984 to 2017, RA diagnosis was based on the following criteria: the 1958 American Rheumatism Association (ARA) criteria [62], the 1987 ARA criteria [63], the eighth revision of the International Classification of Diseases (ICD-8) [64], and the 2010 ACR/EULAR RA classification criteria [65]. Additionally, the cohorts included in these studies may have been affected by several potential confounding factors, including participants’ socioeconomic status, ethnicity, and history of breastfeeding (either prior or concurrent). Finally, definitions of OC use varied across the included studies, potentially contributing further to heterogeneity in our meta-analysis.

We are aware of certain limitations in our study, stemming from the limited number of prospective studies available in the literature, the heterogeneity in study designs, the small sample sizes across enrolled patients, and the variability in the definition of RA criteria. Additionally, the lack of stratification by oral contraceptive formulation represents a further limitation, as it was not possible to assess the differential effects of combined estrogen–progesterone versus progesterone-only preparations on the immune system and RA disease activity due to insufficient data. These variations contribute to the substantial variability observed in our results.

Nevertheless, our current study’s findings seem more robust than three previously published meta-analyses. Firstly, our meta-analysis incorporated more research studies (22), encompassing a larger cohort of RA cases (1290 participants). Secondly, the discrepancies in the results from prior meta-analyses were primarily attributed to varying selection criteria in the original studies and different effect sizes associated with distinct OC use statuses [12,13,14]. In our study, we implemented a more stringent selection criterion and computed the summary odds ratio (OR) across all patient cohorts.

Furthermore, the extended time frame and inclusion of recent studies provide a more comprehensive understanding of the potential protective role of OCs. It is essential to consider the advancements in diagnostic criteria and the improved accuracy in identifying RA cases in recent years, which likely contribute to the observed protective association. The findings regarding both current and past use of oral contraceptives remain inconclusive due to limited evidence and substantial heterogeneity among studies; therefore, further high-quality studies, particularly randomized controlled trials, are warranted to clarify these associations.

Future research should focus on large-scale, well-designed prospective studies to validate these findings and explore the underlying biological mechanisms. Additionally, investigating the impact of different formulations and durations of OC use could offer more nuanced insights into their role in modulating RA risk.

## Data Availability

No new data were created or analyzed in this study.

## Supplementary Materials

The following supporting information can be downloaded at https://www.mdpi.com/article/10.3390/jcm14082710/s1: Table S1: PRISMA checklist; Table S2: MOOSE (Meta-analyses Of Observational Studies in Epidemiology) Checklist; Publication bias; Trim-and-Fill_Results. References [66,67] are cited in the Supplementary Materials.

## Abbreviations

The following abbreviations are used in this manuscript:

RA	Rheumatoid arthritis
OCs	Oral contraceptives
PRISMA	Preferred Reporting Items for Systematic Reviews and Meta-Analyses
MOOSE	Meta-Analyses and Systematic Reviews of Observational Studies
PRISMA-P	Preferred Reporting Items for Systematic Reviews and Meta-Analyses Protocol
MedLine	Medical Literature Analysis and Retrieval System Online
OCs	Oral contraceptives
HRT	Hormone replacement therapy
PICO	Population, Intervention, Comparison, Outcome
OR	Odds ratio
CI	Confidence interval
NOS	Newcastle–Ottawa Quality Assessment Scale
US	United States
RStudio	Integrated Development Environment for R
I^2^	I-squared (statistical measure of heterogeneity)
DBA/1	mice (a strain genetically prone to developing collagen-induced arthritis)
CII	Type II Collagen
CR1	Complement Receptor 1
OPG	Osteoprotegerin
BMP	Bone Morphogenetic Protein
Th1/Th2	T helper type 1 and type 2 cell
Treg	Regulatory T cells
ST6GAL1	Beta-galactoside alpha-2,6-sialyltransferase 1
IL-6	Interleukin-6
B220 + CR1 +	Complement receptor 1 B-cells
M-CSF	Macrophage Colony-Stimulating Factor
RANKL	Receptor Activator of Nuclear Factor κB Ligand
Wnt signaling	Wingless/Integrated signaling pathway
